# Caregivers’ Knowledge, Attitudes, and Practices in terms of Oral Care Provided to Children with Autism Spectrum Disorder

**DOI:** 10.3390/healthcare13131563

**Published:** 2025-06-30

**Authors:** Susana Beatriz Esparza Loredo, Guadalupe Silvia García De la Torre, María Del Carmen Villanueva Vilchis, Saray Aranda Romo, Fátima del Carmen Aguilar Díaz

**Affiliations:** 1 Department of Oral Public Health, National School of Higher Studies (ENES)-Leon, National Autonomous University of Mexico, Leon 37684, Guanajuato, Mexico; susan_eslo@hotmail.com (S.B.E.L.); cvillanueva@enes.unam.mx (M.D.C.V.V.); 2 Dental Science, Master’s and Doctoral Program in Medical, Dental and Health Sciences, National Autonomous University of México, Coyoacan, Mexico City 04510, Mexico; 3 ^3^ Department of Public Health, Faculty of Medicine, National Autonomous University of Mexico, Coyoacan, Mexico City 04360, Mexico; ggartorr@unam.mx; 4 Diagnostic Clinic, Faculty of Stomatology, Autonomous University of San Luis Potosi, San Luis Potosi 78290, San Luis Potosi, Mexico; sarayaranda@fest.uaslp.mx

**Keywords:** autism spectrum disorder, autistic disorder, autism, knowledge, practice, oral care, caregivers, parents, characteristics, sex

## Abstract

**Background/Objectives:** Children with Autism Spectrum Disorder (ASD) often exhibit similar food-related behaviors, such as excessive sugar consumption, and sensory processing difficulties, which can hinder oral hygiene routines like toothbrushing and increase the risk of cavities or gum problems. Therefore, caregiver involvement in maintaining oral health is crucial. The aim of this study was to assess the knowledge, attitudes, and practices reported by caregivers in terms of oral care provided to children diagnosed with ASD between the ages of 5 and 12 years. **Methods:** A cross-sectional study was conducted, and the participants comprised 72 caregivers of children with ASD enrolled in four therapeutic centers in SLP, Mexico. Data on caregivers’ knowledge, attitudes, and care in terms of oral health, as well as sociodemographic characteristics, were collected through a structured and self-administered questionnaire. **Results:** Among the caregivers, 85% were women, and 86% recognized sugar as a cariogenic and gingival bleeding as a sign of inflammation. Despite this, over 60% reported frequent sugar consumption, 65.4% supervised toothbrushing, and floss use was minimal. More than half showed high self-efficacy, which correlated with more frequent supervised or autonomous toothbrushing. Caregivers involved in homecare brushed their children’s teeth more often. Correct knowledge of brushing frequency was associated with actual toothbrushing practices (*p* < 0.05). **Conclusions:** This study highlights a notable gap between caregivers’ knowledge and oral care practices in terms of children with ASD.

## 1. Introduction

Autism Spectrum Disorder (ASD) is a neurodevelopmental condition characterized by persistent difficulties in social interaction and communication, as well as restricted interests and stereotyped activities [1,2,3]. Recent global estimates indicate a prevalence of approximately 1% of children being diagnosed with ASD [4,5]; in Mexico, in 2013, a prevalence of approximately 1 in every 115 children was reported [6].

Children with ASD are considered more vulnerable to oral health problems, which is probably associated with sensory integration disorder [7,8], which involves atypical sensory processing [9]. This condition can interfere with the establishment of self-care routines, such as toothbrushing, increasing the risk of alterations in the oral microbiota and subsequent periodontal disease [7,10,11]. In addition, atypical eating behaviors, including food selectivity, preferences, limited intake, or aversion to specific food textures [9], along with cariogenic dietary habits (frequent consumption of sweets, soft drinks, and sugary snacks) [12], further increase the risk of dental caries in this population [10,11,13].

Maintaining adequate hygiene in children with ASD is, therefore, essential for their overall health and well-being [13]. During early childhood, caregivers play a central role in the daily care and management of children with ASD [4]. Their involvement is closely linked to their knowledge and attitudes about both the disorder and the specific support needs of their children [14].

According to the conceptual model of stigma, there is an interconnection between knowledge, attitudes, and behaviors. A caregiver’s lack of knowledge can lead to negative attitudes (prejudice) and inadequate behaviors [15]. Conversely, an increased awareness and understanding of ASD and its implications for daily care may foster positive caregiving attitudes, particularly regarding health and personal hygiene [16].

Previous research has documented an association between caregivers’ knowledge and children’s oral health, regardless of the underlying condition [17]. However, many caregivers remain unaware of adequate oral health practices [18], often relying on informal or inappropriate sources of information, such as family advice, cultural norms, or practices shaped by sociodemographic variables [14,19]. Factors such as maternal age, marital status, or the child’s birth order have also been linked to caregivers’ attitudes and oral health-related behaviors [14,20,21].

Attitudes toward oral hygiene are reflected in caregiving practices: a positive attitude is associated with better oral health outcomes in children [18], while a negative or indifferent attitude may contribute to poor oral health, especially in children with ASD [19].

Despite the relevance of caregivers in promoting oral hygiene in children with ASD, existing data on their knowledge, attitudes, and practices remain limited. Therefore, this study aimed to evaluate the knowledge, attitudes, and oral health care practices of caregivers in terms of children with ASD between 5 and 12 years of age, enrolled in therapeutic centers in the city of SLP, Mexico, during 2022 and 2023. The goal is to provide a situational diagnosis that informs future intervention strategies to improve oral hygiene in this vulnerable population.

## 2. Materials and Methods

### 2.1. Study Design

This is a cross-sectional study that was approved by the Ethics and Research Committee of the National School of Higher Studies (ENES), Leon (CEI-21-06-S17), and it received permission from the directors of the therapeutic centers selected.

### 2.2. Study Population

This study included the primary caregivers of children aged 5 to 12 years with an ASD diagnosis attending therapy or care centers in San Luis Potosi, SLP, Mexico (Cielo Azul, Centro Educativo País de las Maravillas, Manuel López Dávila, and Fundación SAYA), between 2022 and 2023.

### 2.3. Data Collection Procedures

Participants were selected based on the following inclusion criteria: caregivers of a child with a confirmed diagnosis of ASD (according to the DSM-V criteria) [2], over 18 years of age, residing in SLP, and regularly attending one of the selected therapeutic centers. Participation required the acceptance of informed consent. Caregivers with prior training in oral health were excluded.

The therapeutic centers were randomly selected from the list of those belonging to a Foundation A.C. Invitation to participate was given virtually and in person to all attendees at the centers during July 2023.

### 2.4. Measurements Tools

Data were collected using a structured, self-administered questionnaire composed of five sections:

(1) Caregiver demographics: sex, age, educational level, marital status, and number of children.
(2) Child characteristics: sex, age, and ASD severity levels.
(3) Knowledge: 12 multiple-choice signing questions on oral hygiene, caries, biofilm, caries, and periodontal disease [22].
(4) Attitudes: 15 items on the importance of oral health in childhood [18]. A total of 8 items assessed self-efficacy using a 3-point Likert scale (0–24 points). The score was dichotomized from the median: high self-efficacy 19–24 points; low self-efficacy 0–18 points. The other 7 items on perceived difficulties or concerns related to oral care were also evaluated on a 3-point Likert scale (0–21 points). The score was dichotomized from the median: low difficulty (0–12 points) and high difficulty (13–21 points).
(5) Oral hygiene practices and diet: 4 questions regarding brushing, frequency, flossing, and sugar consumption).

The questionnaire was adapted from previously validated instruments [18,22] and modified to ensure cultural relevance in our population. The instrument underwent both face and cultural validation by four experts: a pediatric dentist, two dentists, and a psychotherapist specializing in ASD. Pilot tests were conducted with 20 caregivers, who were excluded from the main study sample.

### 2.5. Study Size

The sample size was calculated using the formula for finite populations, considering a 95% confidence level (Z = 1.96), a 5% margin of error (e = 0.05), and an expected proportion of 50% (*p* = 0.5) to ensure maximum variability. Considering the limited number of children enrolled at each center—approximately 25 per center—the total available population was estimated at *n* = 100. Based on these parameters, the minimum required sample size was calculated to be 80 caregivers. Ultimately, 72 caregivers participated in this study, representing a high response rate and providing adequate statistical power for the analysis conducted.

### 2.6. Statistical Analysis

The data were entered and analyzed using SPSS (version 23.0). Descriptive statistics, including the means and standard deviations, were calculated for the quantitative variables, while frequencies and percentages were used for the qualitative data. The Shapiro–Wilk tests was applied to assess the normality of the quantitative variables.

The chi^2^ test was used to assess the association between ASD severity levels and oral hygiene practices, as well as between caregivers’ knowledge and attitudes and those practices. Fisher’s exact test was applied to assess the association between caregivers’ attitudes and practices regarding oral health and their demographic and socioeconomic characteristics. The significance level was estimated at *p* < 0.05.

## 3. Results

Seventy-two primary caregivers of children with ASD participated in the study. The majority were female (84.7%, *n* = 61), with a mean age of 36.1 (±7.1; range: 24–52 years). More than half (57%) reported being married or in free union. Regarding occupation, almost 40% (*n* = 28) were housewives, and slightly more than 60% reported being employed. A total of 45% had a higher education level and the rest were distributed between elementary and middle-upper levels. Seventy percent of the caregivers reported having one or two children at most. The results obtained of the sociodemographic variables are shown in Table 1.

Most of the children with ASD were male (63.8%) (2:1 ratio). The average age was 7.85 (±1.8) years, with a range from 5 to 12 years. Regarding ASD severity levels, 58.3% were classified as Level I (requiring support to adapt socially and manage changes, while maintaining a higher degree of independence), whereas 30.6% were categorized as Level II (requiring more substantial support, particularly in communication and coping with changes due to more noticeable social difficulties and repetitive behaviors).

In terms of caregivers‘ oral health knowledge, over 86% correctly identified that sugar consumption is associated with caries development and that gum bleeding is a sign of inflammation, often resulting from infrequent toothbrushing. Nearly 70% recognized the importance of brushing/flossing in preventing gingivitis. Additionally, 34.7% accurately identified the concept of dental biofilm or plaque. However, only 8.3% of caregivers correctly reported the recommended frequency of toothbrushing (three times per day), and 23.6% knew the advised brushing duration (approximately two minutes). Almost 50% the appropriate amount of toothpaste and the recommended toothbrush replacement interval, while 56.9% indicated that dental check-ups should occur every six months.

**Figure 1:**
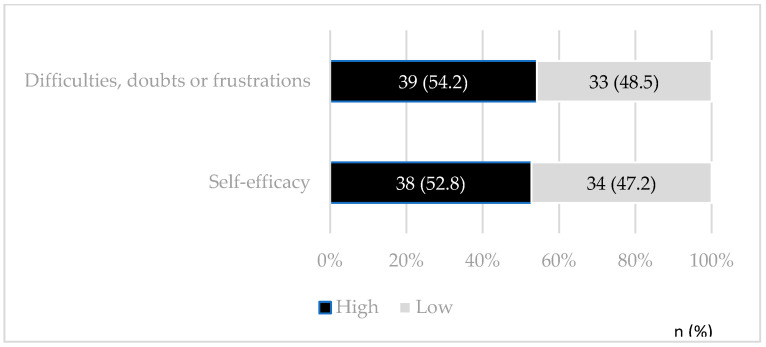
Caregivers’ attitudes toward oral health in children with ASD. S.L.P., S.L.P. 2023, *n* = 72.

**Figure A1:**
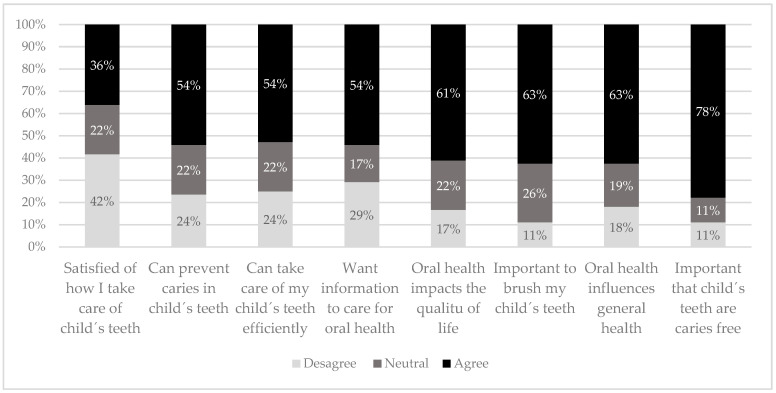
Caregivers’ perceptions of self-efficacy in managing ASD children’s oral care based on Likert scale. S.L.P., S.L.P. 2023, *n* = 72.

**Figure A2:**
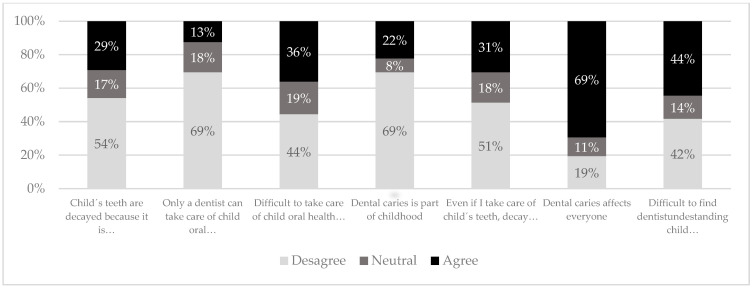
Caregivers’ difficulties or concerns of ASD children’s oral care based on Likert scale, S.L.P., S.L.P. 2023, *n* = 72.

Figure 1 shows the results related to caregivers’ attitudes. Slightly more than half (52%) demonstrated a high level of self-efficacy regarding oral care. Furthermore, 54% were rated as having a high level of concern or awareness about potential difficulties. A detailed item-by-item breakdown of attitude responses is provided in Figure A1 and Figure A2.

Regarding the evaluation of oral care practices, as shown in Table 2, more than one-third of the caregivers reported that the children consume sugary foods “always” or “sometimes”. In addition, nearly 20% indicated that their children never brush their teeth. Among those who reported that their children do engage in toothbrushing, 65.3% stated that it is performed with the help and/or supervision of an adult. However, only 4.2% reported the use of oral hygiene accessories, such as dental floss. None of these practices were found to be associated with the severity levels of ASD in the children.

None of the sociodemographic characteristics of the caregivers were significantly associated with knowledge and attitudes (*p* > 0.05). The results are shown in Table 3.

On the other hand, some of the oral hygiene practices were associated with sociodemographic characteristics, such as caregivers’ educational level and children’s sugar consumption (*p* < 0.05). Additionally, a higher proportion of caregivers dedicated to homecare reported brushing their children’s teeth more frequently compared to those who were employed (Table 4).

Finally, the association between knowledge and attitudes and practices were examined in relation to sugar consumption, toothbrushing frequency, and the use of dental floss (Table 5). A significantly greater proportion of caregivers who correctly identified recommended toothbrushing frequency reported brushing their children’s teeth twice or more per day (*p* = 0.016). With respect to attitudes reflecting active caregiver involvement, more than half of those with high self-efficacy scores reported supervising their children’s toothbrushing (55%), and 90% reported that their children brushed autonomously (*p* = 0.001).

## 4. Discussion

This study is among the first to identify the oral care practices performed in Mexican children with ASD aged 5 and 12 years, as well as to evaluate the knowledge and attitudinal disposition of their primary caregivers regarding oral hygiene.

Evidence suggests that children under 12 often lack adequate toothbrushing skills [23], which may be attributed to limited dexterity, inadequate understanding, or low motivation [24,25]. Therefore, this study focused on the caregivers of children under this age threshold. In children with ASD, these challenges are further compounded by oral defensiveness—an aversive reaction to intraoral or perioral stimuli due to hypersensitivity— which complicates the establishment of consistent oral hygiene routines [26,27].

In this context, parents or caregivers play a crucial role, not only in supporting the child’s daily needs but also in facilitating oral care, as children with ASD frequently require continuous and long-term assistance to manage both behavioral and sensory challenges [4].

### 4.1. Knowledge

Caregivers in this study demonstrated adequate awareness of the relationship between sugar intake and caries development. However, a considerable proportion still reported frequent sugar consumption by their children, suggesting a disconnect between knowledge and practice. This inconsistency may be explained by the inflexible routines and restrictive eating behaviors that characterize ASD [9,12].

Most caregivers recognized toothbrushing as a key preventive measure against periodontal problems but lacked understanding of the role of biofilm accumulation in disease progression. This finding aligns with Piraneh H, who reported similar gaps in knowledge [28]. Murshid [29] identified inadequate toothbrushing as the primary cause of caries in children with ASD. Additionally, Floríndez [30], reported that Latino parents perceive the condition itself as a barrier to successful oral hygiene practices.

### 4.2. Attitudes

Currently, the literature on caregiver attitudes toward oral care in children with ASD is heterogeneous, limiting direct comparisons across studies. It is important to note that certain types or components of attitudes may be more predictive of behavior than others [31]. In this study, caregivers scored higher on items related to self-efficacy, suggesting a generally positive disposition toward oral care tasks. These findings are consistent with Alqahtani AS, who found that nine out of ten caregivers believed oral health to be important and influential to the overall well-being of children with ASD [32].

In ideal scenarios, knowledge about oral health influences caregiver’s attitudes and, subsequently, their behavior [15]. This pattern was observed by AlHumaid J [18], who reported that caregivers with more positive attitudes were associated with better oral health status and improved dietary habits among their children. However, these outcomes were not evaluated in our population.

### 4.3. Practices

A relevant finding of this study is that most children brushed their teeth regularly, usually with caregiver supervision. Despite widespread recognition of toothbrushing as a preventive strategy, the use of dental floss and rinses was minimal. This may be due to caregivers’ lack of knowledge or concerns about potential ingestion [33,34].

Caregivers’ sociodemographic characteristics appear to influence oral hygiene practices [14,20]. In our sample, children whose caregivers had lower educational levels were more likely to consume sugary foods frequently. As noted by Guralnick [35], caregiver educational level and occupation serve as distal factors that reflect broader developmental influences in children with ASD. In this context, health literacy may act as a mediating factor. Limited health literacy, often associated with lower educational levels, can hinder caregivers’ ability to make informed decisions, contributing to suboptimal dietary and hygiene practices [36].

Toothbrushing frequency was higher among children whose caregivers were dedicated to homecare. This may be explained by the increased time and attention that these caregivers are able to devote to daily routines [37,38]. Frequently, this role is assumed by women who leave paid employment to focus entirely on caregiving. Although this dedication may benefit oral care, it often comes at the cost of emotional and physical well-being. Role overload can lead to stress, anxiety, and caregiver burnout, which may in turn compromise the consistency and quality of oral hygiene practices [39,40].

This study has several limitations. These include a limited sample size, the use of self-report measures to assess some variables, and the exclusion of key behavioral aspects, such as toothbrushing technique, use of mouthwash, and clinical oral health indicators. Furthermore, due to its cross-sectional and quantitative design, this study is limited to exploratory associations and does not allow for causal inferences.

Another potential limitation is social desirability bias, as data were collected through self-reported questionnaires. Caregivers may have responded in a way they believed to be socially acceptable or expected. This bias could lead to overestimation of positive behaviors (frequency of toothbrushing or supervision) and underreporting of less favorable practices.

This study identifies part of the practices for oral health care of children with ASD. However, additional data sources, such as video recordings of toothbrushing routines or clinical oral assessments, could provide deeper insights into the specific challenges faced by caregivers.

Among the strengths, we can consider that this is a pioneer study regarding oral care in the Mexican population of children with ASD. To improve the quality and depth of future research, it is recommended to incorporate longitudinal designs that allow for observations of changes over time and help establish causal relationships. Additionally, integrating educational interventions to address caregivers’ understanding of the autism condition and its specifical impact on oral health could provide a more contextualized perspective. These approaches would not only strengthen data viability but also support the development of tailored oral health strategies for ASD children.

In this context, the active involvement of pediatric dentists is essential not only for individualized clinical care, but also for developing tailored prevention strategies. A multidisciplinary approach involving dental professionals, therapists, and health educators can significantly contribute to improving oral health outcomes and quality of life for ASD children and their families.

## 5. Conclusions

Caregivers’ knowledge regarding oral care for children with ASD was found to be variable and, in some cases, ambiguous. However, their attitudes represent a valuable opportunity to improve oral hygiene practices, particularly given the continuous daily support these children require. Future interventional studies are necessary to address the gaps between limited knowledge and negative attitudes and inadequate oral care. Targeting caregivers’ intrinsic factors, such as motivation, self-efficacy, beliefs, and emotional burden, may strengthen their sense of empowerment and enhance their capacity to support both the oral health and overall well-being of children with ASD.

## Figures and Tables

**Table 1:** Demographic and socioeconomic characteristics of caregivers of children with ASD. S.L.P., S.L.P. 2023.

		*n =* 72
		***n (%)***
**Sex**	Male	11 (15.3)
	Female	61 (84.7)
**Educational level**	Basic	24
	Middle-upper	21 (29.2)
	Bachelors or postgraduate	27
**Occupation**	Home	28 (38.9)
	Work	44 (61.1)
**Marital status**	Single/separated/divorced	31 (43.1)
	Married/free union	41 (56.9)
**Number of children**	Two or fewer	51 (70.8)
	Three or more	21 (29.2)

**Table 2:** Self-reported oral health practices of caregivers of children with ASD. S.L.P.; S.L.P. 2023.

		ASD Severity Levels Of Children				
		Requiring Support	Requiring Substantial Support	Requiring Substantial Support	Total *n (%)*	*p* *
**Sugar consumption**	Never	1 (2.4)	1 (4.5)	0	2 (2.8)	0.223
	Sometimes	14 (33.3)	7 (31.8)	6 (75)	27 (37.5)	
	Always	27 (64.3)	14 (63.6)	2 (25)	43 (59.7)	
	*Total*	*42 (100)*	*22 (100)*	*8 (100)*	*72 (100)*	
**Frequency of toothbrushing**	No	7 (16.7)	7 (31.8)	0	14 (19.4)	0.159
	Once	16 (38.1)	10 (45.5)	3 (37.5)	29 (40.3)	
	Twice or more	19 (45.2)	5 (22.7)	5 (62.5)	29 (40.3)	
	*Total*	*42 (100)*	*22 (100)*	*8 (100)*	*72 (100)*	
**Toothbrushing practice**	No	10 (23.8)	4 (18.2)	0	14 (19.4)	0.381
	Yes, on his/her own	8 (19)	2 (9.1)	1 (12.5)	11 (15.3)	
	Yes, with supervision	24 (57.1)	16 (72.7)	7 (87.5)	47 (65.3)	
	*Total*	*42 (100)*	*22 (100)*	*8 (100)*	*72 (100)*	
**Use of dental floss**	No	40 (95.2)	21 (95.5)	8 (100)	69 (95.8)	0.813
	Yes, on his/her own	0	0	0	0	
	Yes, with supervision	2 (4.7)	1 (4.5)	0	3 (4.2)	
	*Total*	*42 (100)*	*22 (100)*	*8(100)*	*72 (100)*	

*p* * = Chi^2^ test.

**Table 3:** Caregivers’ attitudes toward oral health according to sociodemographic characteristics. S.L.P., S.L.P. 2023, *n* = 72.

		Sex			Educational Level				Occupation			Marital Status			Number of Children			*Total* *n (%)*	
		Male	Female	p *	Basic	Middle Upper	Bachelors/ Postgraduate	p *	Home	Work	p *	Single/separated/divorced	Married /free union	p *	Two or less	Three or more		p *	
**Self-efficacy**	High	7 (63)	31 (49)	0.522	14 (58)	11 (52)	13 (48)	0.828	13 (46.4)	25 (56.8)	0.472	15 (48)	23 (56)	0.635	26 (51)	12 (57)		0.796	38 (52.7)
	Low	4 (37)	30 (51)		10 (42)	10 48)	14 (52)		15 (53.6)	19 (43.2)		16 (52)	18 (44)		25 (49)	9 (43)			34 (47.2)
	***Total***	11 (100)	61 (100)		24 (100)	21 (100)	27 (100)		28 (100)	44 (100)		31(100)	41 (100)		51 (100)	21 (100)			72 (100
**Difficulties or doubts**	High	5 (45.5)	34 (55.7)	0.744	12 (50)	14 (67)	13 (48)	0.721	19 (32)	20 (45.5)	0.090	16 (52)	23 (56)		0.812	25 (49)	14 (67)	0.202	39 (54.1)
	Low	6 (54.5)	27 (44.3)		12 (50)	7 (33)	14 (52)		9 (68)	24 (54.5)		15 (48)	18 (43)			26 (51)	7 (33)		33 (45.8)
	***Total***	11 (100)	61 (100)		24 (100)	21 (100)	27 (100)		28 (100)	44 (100)		31 (100)	41 (100)			51 (100)	21 (100)		72 (100)

*p* * = Fisher’s exact test.

**Table 4:** Association between caregivers’ sociodemographic characteristics and reported oral hygiene practices in children with ASD. S.L.P., S.L.P. 2023, *n* = 72.

		Sex *n (%)*			Educational Level *n (%)*				Occupation *n (%)*			Marital Status *n (%)*			Number of Children *n (%)*		
		Male	Female	p *	Basic	Middle-upper	Bachelors/postgraduate	p *	Home	Work	p *	Single/separated/divorced	Married/free union	p *	2 or fewer	3 or more	p *
**Sugar consumption**	**Always**	5 (45.5)	22 (36.1)	0.405	4 (16.7)	10(47.6)	13(48.1)	0.027	20 (71.4)	23 (52.3)	0.216	20 (64.5)	23 (56.1)	0.723	28 (54.9)	15 (71.4)	0.344
	**Sometimes**	6 (55.5)	37 (60.7)		20 (83.3)	11 (52.4)	12 (44.4)		7 (25)	20 (45.5)		10 (32.3)	17 (41.5)		21 (41.2)	6 (28.6)	
	**Never**	0	2 (3.3)		0	0	2 (2.8)		1 (3.6)	1 (2.3)		1 (3.2)	1 (2.4)		2 (3.9)	0	
**Toothbrushing practice**	**No**	5 (45.5)	38 (62.3)	0.396	3 (12.5)	6 (28.6)	5 (18.5)	0.911	4 (14.3)	10 (22.7)	0.384	7 (22.6)	7 (17.1)	0.486	12 (23.5)	2 (9.5)	0.063
	**Yes, on** **his/her own**	6 (54.5)	21 (34.4)		5 (20.8)	2 (9.5)	4 (14.8)		3 (10.7)	8 (18.2)		3 (9.7)	8 (19.5)		10 (19.6)	1 (4.8)	
	**Yes, with supervision**	0	2 (3.3)		16 (66.7)	13 (61.9)	18 (66.7)		21 (75)	26 (59.1)		21 (67.7)	26 (63.4)		29 (56.9)	18 (85.7)	
**Toothbrushing frequency**	**No**	3 (27.3)	11 (18)	0.776	4 (16.7)	2 (9.5)	8 (29.6)	0.257	2 (7.1)	12(27.3)	0.050	9 (29)	5 (12.2)	0.176	12 23.5)	2 (9.5)	0.378
	**Once**	4 (36.4)	25 (41)		7 (29.2)	10 (47.6)	12 (44.4)		11(39.3)	18 (40.9)		12 (38.7)	17 (41.5)		19 (37.3)	10 (47.6)	
	**Twice or more**	4 (36.4)	25 (41)		13 (54.2)	9 (42.8)	7 (25.9)		15 (53.6)	14 (31.8)		10 (32.2)	19 (46.3)		20 (39.2)	9 (42.8)	

*p* * = Chi^2^ test.

**Table 5:** Association between caregivers’ knowledge and attitudes and oral hygiene practices in children with ASD. S.L.P., S.L.P. 2023.

*n* = 72 *n (%)*														
			**Knowledge**								**Attitudes**			
			**Proper Identification of Sugar Consumption Associated with Dental Caries**		**Adequate Identification of Brushing Frequency (Twice or More Times Daily**		**Recognition of the Importance of Brushing/Flossing**		**Recognition of Gingivitis as a Sign of Inflammation**		**High Self-Efficacy**		**High Difficulty or Frustration**	
			***n* (%)**	*p* *	***n (%)***	*p* *	***n (%)***	*p* *	***n (%)***	*p* *	***n (%)***	*p* *	***n (%)***	*p* *
**Practices**	**Sugar consumption**	Never	2 (3.2)	0.157	1 (1.6)	0.304	1 (2.6)	0.701	2 (92.6)	0.240	2 (100)	0.232	1 (50)	0.711
		Sometimes	36 (58.1)		38 (61.3)		24 (63.2)		41 (95.3)		20 (46.5)		25 (58)	
		Always	24 (38.7)		23 (37.1)		13 (34.2)		25 (100)		16 (59)		13 (48)	
	**Toothbrushing frequency**	No	13 (92.9)	0.392	9 (64.3)	0.016	5 (35.7)	0.861	14 (100)	0.311	4 (28.6)	0.113	5 (35.7)	0.304
		Once	22 (75.9)		25 (86.2)		17 (58.5)		28 (96.6)		16 (55)		17 (58.6)	
		Twice or more	27 (93.1)		28 (96.6)		16 (55.2)		26 (89.7)		18 (62)		17 (58.6)	
	**Toothbrushing practice**	No	14 (22.6)	0.620	11 (17.7)	0.629	4 (10.5)	0.580	14 (100)	0.324	2 14.3)	0.001	6 (42.9)	0.630
		Yes, on his/her own	10 (16.1)		10 (16.1)		7 (18.4)		11 (100)		10(90.9)		6 (54.5)	
		Yes, with supervision	38 (61.3)		41 (66.1)		27 (71.1)		43 (91.5)		26(55.3)		27 (57.4)	
	**Use of dental floss**	No	60 (87)	1.00	59 (85.5)	0.905	35 (50.7)	0.793	65 (94.2)	0.796	37 (53.6)	0.632	39 (56.5)	0.641
		Yes, on his/her own	---		---		---		---		--		--	
		Yes, with supervision	2 (66.7)		3 (100)		3 (100)		3 (100)		1 (33.3)		--	

*p* * = Fisher exact test.

## Data Availability

The datasets used and/or analyzed during the current study are available from the corresponding author upon reasonable request.

## Abbreviations

ASD	Autism Spectrum Disorder
SLP	San Luis Potosí, México
DSM-V	Diagnostic and Statistical Manual of Mental Disorders
ENES	Escuela Nacional de Estudios Superiores
UNAM	Universidad Nacional Autónoma de México

## Appendix A

Caregivers’ attitudes toward ASD children’s oral care.
